# A review of study designs and statistical methods for genomic epidemiology studies using next generation sequencing

**DOI:** 10.3389/fgene.2015.00149

**Published:** 2015-04-20

**Authors:** Qian Wang, Qiongshi Lu, Hongyu Zhao

**Affiliations:** ^1^Program of Computational Biology and Bioinformatics, Yale UniversityNew Haven, CT, USA; ^2^Department of Biostatistics, Yale School of Public HealthNew Haven, CT, USA; ^3^Veterans Affairs Cooperative Studies Program Coordinating CenterWest Haven, CT, USA

**Keywords:** next-generation sequencing, genomic epidemiology, study design, statistical methods, genetic etiology

## Abstract

Results from numerous linkage and association studies have greatly deepened scientists’ understanding of the genetic basis of many human diseases, yet some important questions remain unanswered. For example, although a large number of disease-associated loci have been identified from genome-wide association studies in the past 10 years, it is challenging to interpret these results as most disease-associated markers have no clear functional roles in disease etiology, and all the identified genomic factors only explain a small portion of disease heritability. With the help of next-generation sequencing (NGS), diverse types of genomic and epigenetic variations can be detected with high accuracy. More importantly, instead of using linkage disequilibrium to detect association signals based on a set of pre-set probes, NGS allows researchers to directly study all the variants in each individual, therefore promises opportunities for identifying functional variants and a more comprehensive dissection of disease heritability. Although the current scale of NGS studies is still limited due to the high cost, the success of several recent studies suggests the great potential for applying NGS in genomic epidemiology, especially as the cost of sequencing continues to drop. In this review, we discuss several pioneer applications of NGS, summarize scientific discoveries for rare and complex diseases, and compare various study designs including targeted sequencing and whole-genome sequencing using population-based and family-based cohorts. Finally, we highlight recent advancements in statistical methods proposed for sequencing analysis, including group-based association tests, meta-analysis techniques, and annotation tools for variant prioritization.

## Introduction

**FIGURE 1:**
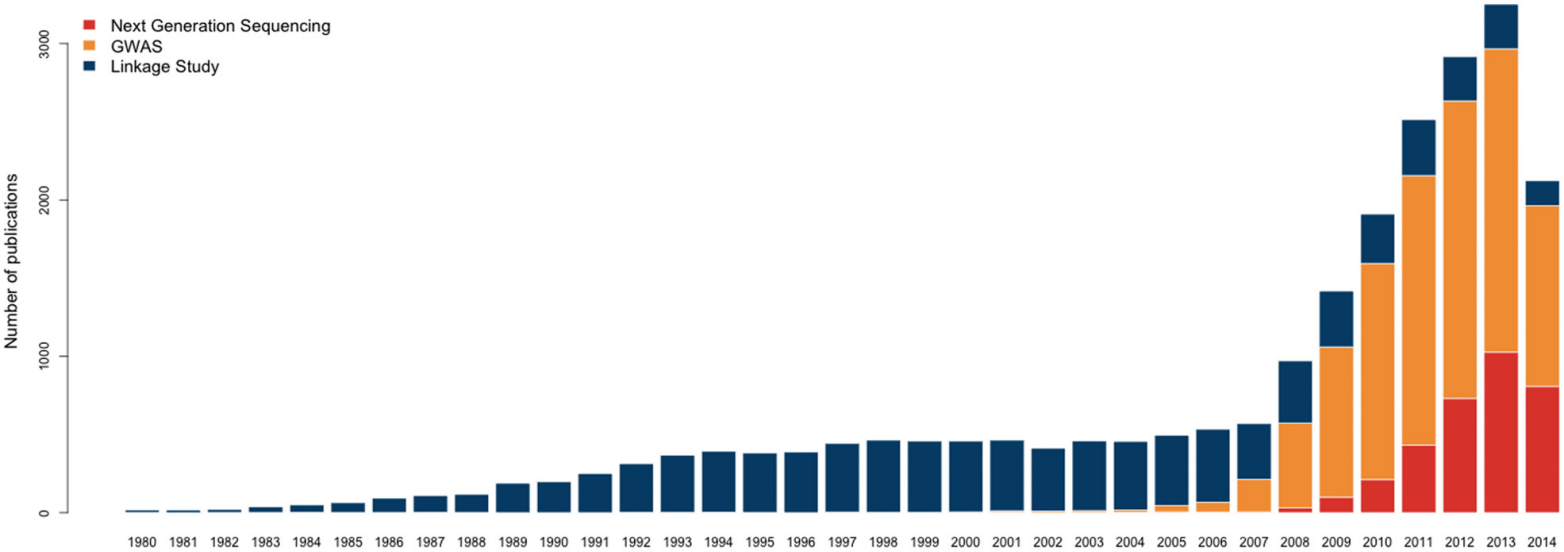
**Number of publications studying human traits using various techniques (1980–2014).** Only the publications in core clinical journals or MEDLINE are included. The vertical axis indicates the number of publications and the horizontal axis indicates different years. The three colors correspond to genetic linkage studies, GWAS, and next generation sequencing, respectively.

The rapid advancement of biotechnology has brought paradigm shifts in genetic/genomic epidemiology. From linkage studies to genome-wide association studies (GWAS) to the extensive application of next-generation sequencing (NGS), technological developments have improved study designs, deepened our understanding of disease etiology, and led to numerous scientific discoveries (**Figure 1**). This can be seen in the study of Crohn’s disease, an inflammatory bowel disease with prevalence 0.32% in Europe and North America (Molodecky et al., 2012). Twin-based epidemiological analysis first suggested that there is a genetic component of Crohn’s disease (Molodecky et al., 2012); family-based linkage studies then identified six loci associated to the disease (Hugot et al., 1996; Cardinale et al., 2013); GWAS identified 163 loci at genome-wide significance level, which collectively explain 13.6% of the phenotypic variance (Duerr et al., 2006; Imielinski et al., 2009; Jostins et al., 2012); and re-sequencing GWAS loci identified several causal variants with low minor allele frequencies (Momozawa et al., 2011; Rivas et al., 2011; Cardinale et al., 2013; Ellinghaus et al., 2013; Hunt et al., 2013).

Twin-based epidemiological studies can be used to estimate the broad-sense disease heritability, i.e., the amount of phenotypic variance that can be explained by all genetic factors, through comparing phenotype concordance rate in monozygotic and dizygotic twins (Veale, 1960). Linkage studies, which combine information from family pedigrees and sparse genetic markers, can be used to locate disease-associated loci on a very rough scale (Nance et al., 1969; Greer et al., 1989; St George-Hyslop et al., 1990; Dawn Teare and Barrett, 2005). GWAS benefits from the array technology that allows millions of markers to be genotyped at reasonable cost and speed with high accuracy. It has deepened our understanding of disease etiology in multiple directions. First, since the first successful GWAS was published Klein et al. (2005), GWAS has become widely adopted and led to the identifications of a large number of disease-associated genomic loci. As of February 20, 2015, 15,396 single nucleotide polymorphisms (SNPs) from 2,111 publications have been documented in the GWAS Catalog (Welter et al., 2014; Hindorff et al., 2015). Second, the associated loci discovered from GWAS could serve as risk predictors for some diseases, provided large enough GWAS discovery sample size (Wray et al., 2008). Those genetic risk predictors, either used alone or combined with traditional non-genetic risk prediction, have the potential to improve risk-prediction accuracy, which might benefit clinical diagnose and personalized treatment (Jostins and Barrett, 2011). Third, dense genome-wide markers enable a reasonable approximation of narrow-sense heritability (phenotypic variance explained by additive genetic factors) using chip heritability (phenotypic variance explained by genotyped SNPs; Speed et al., 2012; Furlotte et al., 2014). Moreover, valuable insight has been learned on the genetic etiology of many diseases through analyzing the variance contribution of SNPs from certain genomic regions, pathways, as well as variant groups based on minor allele frequencies (Davis et al., 2013; Zaitlen et al., 2014).

As fruitful as GWAS is, it still leaves many intriguing questions unanswered, top on which are the following two problems. The first problem is the difficulty in interpreting GWAS results. This is partly due to our limited understanding of genomic function, especially for non-coding regions, in which considerably many disease-associated loci have been identified. The correlation structure among neighboring variants, often referred to as linkage disequilibrium (LD), also impacts our ability to interpret the results. In fact, it is the haplotype blocks that a GWAS actually identifies, not the real functional variants (Cooper and Shendure, 2011). The second problem is the missing heritability, usually referring to the large gap between the proportion of the variance explained by significant SNPs identified from GWAS and the estimated narrow-sense heritability from twin and pedigree analysis (Manolio et al., 2009; Witte et al., 2014). This has been partly resolved by estimating the chip heritability using linear mixed models and restricted maximum-likelihood estimation in genome-wide complex trait analysis (Yang et al., 2010, 2011). However, a large part of narrow-sense heritability still remains missing. One explanation is the imperfect LD between tagged SNPs and causal variants. Other potential contributors to missing heritability include small insertions and deletions, large structural variants (SVs; Frazer et al., 2009; Mefford and Eichler, 2009), epigenetic factors, gene-by-gene and gene-by-environment interactions (Frazer et al., 2009), and phantom heritability (Zuk et al., 2012). All these genetic and non-genetic factors may have substantial contributions to the etiology for some diseases and disorders.

The rapidly developing NGS technology promises many opportunities to answer some of these questions. Ultimately, it has the potential to provide further biological insight into disease etiology, which may lead to important clinical applications including disease prevention, diagnosis, and treatment. In this review, we discuss why and how, and to which extent NGS techniques can address the issues mentioned above, i.e., zooming in to identify more disease-associated variants or even the real causal ones, and zooming out to recover the missing heritability. We also summarize study designs, statistical methods for analyzing sequencing data, current findings, and challenges.

## Zooming In and Out: Identification of Causal Variants and Dissection of Disease Heritability

Next-generation sequencing can be used to identify not only single nucleotide variants (SNVs), but also SVs and epigenetic variations. SNVs are the easiest to call from sequencing data compared to other variant types. Many methods have been proposed to call SNVs with high accuracy from NGS data [e.g., GATK (McKenna et al., 2010), cortex (Iqbal et al., 2012), and DISCOVAR (Weisenfeld et al., 2014)]. SVs in the human genome include copy number variants, copy-number neutral (balanced) translocations, and inversions of various sizes. Traditionally, large SVs are studied using cytogenetics (Langer-Safer et al., 1982; Schrock et al., 1996), while small SVs require finer technologies such as array comparative genomic hybridization (CGH; de Ravel et al., 2007). A number of methods have also been developed to call SVs from sequencing data, including re-sequencing and *de novo* assembly methods such as MultiBreak-SV (Ritz et al., 2014), GASV (Sindi et al., 2009), LUMPY (Layer et al., 2014), DELLY (Rausch et al., 2012), cn.MOPS (Klambauer et al., 2012), as well as methods reviewed in Medvedev et al. (2009), Tan et al. (2014). Epigenetic variations are the modifications on DNA or chromatin without altering the DNA sequence. Before NGS, detection of epigenetic variations relies on PCR assays (Herman et al., 1996), DNA methylation profiling arrays (Bibikova et al., 2006; Schumacher et al., 2006), and ChIP-chip (Buck and Lieb, 2004). Now, with the help of NGS, epigenetic variations can be detected with ultra high resolution. Moreover, it has become possible to detect allele-specific epigenetic variations through ChIP-seq and bisulfite sequencing (Meissner et al., 2005; Kerkel et al., 2008; Park, 2009; Schalkwyk et al., 2010). Since many types of genomic and epigenetic variations can be detected with improved coverage and accuracy using sequencing data, NGS has the potential to partly recover the missing heritability. Therefore, a more comprehensive view of the decomposition of phenotypic variance is expected from applications of NGS in genetic epidemiology. However, although the variant-calling accuracy of structural and epigenetic variations has been significantly improved, it is still relatively low compared to that of SNVs, especially for earlier sequencing technology with shorter reads. It is also particularly challenging to call SVs using whole-exome sequencing (WES) compared with whole-genome sequencing (WGS). In this review, we focus on the detection of causal SNVs using NGS technology. Issues related to SVs and epigenetic variations can be found elsewhere (Medvedev et al., 2009; Meaburn and Schulz, 2012).

By its study design, GWAS works ideally under the common-disease common-variant (CDCV) hypothesis (Visscher et al., 2012). However, the CDCV hypothesis may not hold for many common diseases as recent studies have suggested a substantial contribution of rare variants to many diseases and traits, as reviewed in Gibson (2012). GWAS will likely fail to detect signals from rare variants in these cases unless the effect size is very large or the causal variants are in strong LD with genotyped markers. With data generated from NGS, researchers can identify more signals in two situations. First, if GWAS does detect the signal (likely to be weak) due to LD structures, the implicated genomic regions can be re-sequenced to uncover the candidates of causal variants. Second, if the disease risk is driven by rare variants independent from the genotyped SNVs, then GWAS will completely miss the signal. In this case, WES or WGS could be used to scan the exome or the genome and detect rare-variant associations in a hypothesis-free manner.

Instead of using LD to detect signals near the probed SNVs, NGS allows researchers to study all the SNVs in each individual directly. However, it will also reveal an overwhelmingly large number of rare variants, most of which have no functional relevance. Therefore, it is non-trivial to identify the causal variants even after accurate variant calling. Moreover, due to the relatively high cost of sequencing compared to other technological options today, most sequencing studies do not have a very large sample size to have adequate statistical power to detect signals through traditional univariate statistical tests. In order to tackle this problem, various strategies in terms of study designs and statistical models have been proposed and implemented. The first strategy is targeted sequencing, including WES and candidate-gene studies. This type of studies usually significantly reduces the cost of sequencing so that larger samples may be analyzed. The second strategy is to focus on a certain type of variants that are more likely to be causal by properly choosing the control group. One example is to use family-trio data to identify *de novo* mutations that only exist in affected children but not in healthy parents (Xu et al., 2011, 2012; Gauglerl et al., 2014; Muona et al., 2014). Finally, statistically sound methods have also been proposed, including group-based association tests (Lee et al., 2014), meta-analysis techniques for sequencing data (Evangelou and Ioannidis, 2013; Lee et al., 2014), and bioinformatics tools for genome annotation (Cooper and Shendure, 2011; Ward and Kellis, 2012b; Hou and Zhao, 2013), to extract most information from the data. These statistical methods are crucial when applying NGS in population-based association studies. All these strategies are discussed below.

## Application of NGS Under Various Study Designs

Next-generation sequencing has brought great success to many different types of studies. First, in terms of cohort type, some studies use family-based data (Vissers et al., 2010; Fromer et al., 2014; Iossifov et al., 2014) while others collect data from unrelated individuals (Hunt et al., 2013; Morrison et al., 2013). Sample sizes are also highly variable in different studies, with extreme cases as small as one individual (Lupski et al., 2010) or as large as tens of thousands subjects (Hunt et al., 2013; De Rubeis et al., 2014). Finally, in terms of sequencing target, WGS, WES, and candidate-gene sequencing all have been applied. Although cost and budget are important factors in choosing a study design, it is crucial to choose the most appropriate study design according to the underlying genetic etiology in order to make meaningful scientific discoveries. For example, tissue-specific diseases may indicate a possible contribution from somatic mutations (Stratton, 2009); diseases that are highly detrimental yet have high prevalence, e.g., autism disorder, may be implicated in causal *de novo* mutations; and common diseases tend to have more complicated genetic etiology than monogenic diseases, usually involving a large number of genetic factors with small effect individually. Prior knowledge of diseases can guide researchers to make the best use of NGS when designing studies. In this section, we review some pioneer research with different study designs.

### Targeted Sequencing and Whole-Genome Sequencing

Due to the still high cost of WGS, targeted sequencing is more commonly used as a cost-effective study design. Popular options include WES using exome-capturing technologies and candidate-gene studies where only a set of preselected genes (e.g., genes close to significant loci identified in GWAS) is sequenced. Although WES misses the entire non-coding genome and sometimes part of the coding regions, numerous scientific discoveries have been made using WES (Majewski et al., 2011). The synthetic association hypothesis (Dickson et al., 2010) provides the theoretical support for re-sequencing GWAS candidate genes in search of causal rare variants. The greater interpretability for variants in the coding regions, the increased statistical power due to less severe multiple testing and the larger sample size due to the much lower cost altogether make WES and candidate-gene studies popular choices.

Re-sequencing GWAS loci of autoimmune diseases have yielded rich positive results (Hunt et al., 2013). By re-sequencing the GWAS significant loci of Crohn’s disease, deleterious or protective variants with low frequencies have been identified in multiple genes, e.g., IL23R and NOD2 (Momozawa et al., 2011; Rivas et al., 2011). By re-sequencing 55 ulcerative colitis GWAS loci in 200 cases and 150 controls, variants with low frequencies were detected in CARD9, IL23R, and RNF186 (Beaudoin et al., 2013). However, re-sequencing 25 GWAS loci in a very large sample (24,892 cases for six autoimmune disease phenotypes and 17,019 controls) did not yield a supportive result for the synthetic association (Hunt et al., 2013). The contribution of rare variants in coding regions is negligible compared with that of common variants. Notably, although this study has a very large sample size, only the exons of 25 GWAS loci were re-sequenced. Therefore, the contribution of rare variants to Crohn’s disease still awaits further assessment with genome-wide screening.

Relatively fewer studies use population-based WGS for complex disorders or traits due to the high cost. However, several pioneer studies have demonstrated the potential of WGS in understanding the genetic architecture of complex diseases, especially for discovering the contribution of rare variants and variants in non-coding regions. For example, the CHARGE consortium (Psaty et al., 2009) performed WGS on 962 individuals to study the levels of high-density lipoprotein cholesterol (HDL-C) and it was found that common (MAF > 0.01) and rare variants (MAF < 0.01) explain about 61.8 and 7.8% of HDL-C level variance, respectively (Morrison et al., 2013). As the sequencing cost continues to drop (now less than $1000 per genome at 30x coverage with the announcement of Illumina Hiseq X Ten system), we can expect more population-based WGS studies in the future. Results from those studies may greatly deepen our understanding of disease architecture, due to their unparalleled coverage of the human genome.

### Rare Diseases and Common Complex Diseases

Next-generation sequencing can be applied to study both rare diseases and common diseases. For rare monogenic diseases, causal variants may be identified even with a small sample size. However, it remains challenging to identify causal alleles for most common diseases as well as some rare diseases with genetic heterogeneity (McClellan and King, 2010), which typically require a larger sample size.

Whole-exome sequencing and WGS allow a revisit to monogenic diseases that are traditionally studied using linkage analysis, and bring the opportunities of finding genetic causes for intractable patients with no previously known causes. Many novel causal variants have been identified, as reviewed in Bamshad et al. (2011), Ku et al. (2011), Dewey et al. (2012). For example, gene *SH3TC2* was found to contain causative alleles for Charcot-Marie-Tooth disease using WGS on one patient (Lupski et al., 2010); WES of four patients identified a causal gene *OHODH* for Miller syndrome (Ng et al., 2010). These results demonstrate the power of NGS in identifying causal variants for monogenic diseases even with very small sample size. There are certain complications, though, caused by potential unnoticed environmental risk factors (Weatherall, 2001) and existence of functionally redundant paralogs of disease genes (Chen et al., 2013).

Not all rare diseases have such a simple genetic structure as monogenic diseases do. Rare diseases are usually diagnosed or defined by symptoms, whereas the same symptom can be induced by different mechanisms. In fact, some rare diseases are a group of diseases manifesting similar symptoms. The identification of causal variants for those diseases generally requires larger sample sizes than monogenic diseases. Muona et al. (2014) recently found a causal *de novo* mutation in gene KCNC1 for progressive myoclonus epilepsy (PME), a group of rare disorders, through WES on 110 unrelated patients (Muona et al., 2014). By doing WGS on 50 patients, Gilissen et al. (2014) discovered major genetic causes for severe and genetically heterogeneous intellectual disability that affects 0.5% of newborns.

Genetic heterogeneity can have many faces for common diseases. Individuals with the same disease may have different causal variants from the same gene or different genes in the disease pathway(s). These variants can be common or rare, coding or non-coding. On the other hand, individuals with the same causal genetic factors may not manifest the same phenotype due to incomplete penetrance, interaction with other genetic, epigenetic, or environmental factors. All of these scenarios may exist simultaneously among patients of a complex disease, making it difficult to characterize the genetic etiology. GWAS can only identify common variants with reasonable effect sizes, which are usually non-causal. NGS may help in this situation as a tool to screen rare variants. One successful application is for neurodevelopmental disorders.

Whole-exome sequencing has achieved great success for *de novo* mutation detection in neurodevelopmental disorders such as autism spectrum disorder (O’Roak et al., 2011, 2012; Iossifov et al., 2012, 2014; Neale et al., 2012; Sanders et al., 2012; Hamilton et al., 2013; De Rubeis et al., 2014; Robinson et al., 2014), mental retardation (Vissers et al., 2010; Gilissen et al., 2014; Wen et al., 2014), and schizophrenia (Awadalla et al., 2010; Girard et al., 2011; Xu et al., 2011, 2012; Fromer et al., 2014; McCarthy et al., 2014; Purcell et al., 2014). Causal *de novo* mutations for these neurodevelopmental disorders are *not* randomly distributed in the genome, as converging evidence has pointed to their enrichment in synaptic, transcriptional and chromatin remodeling genes (De Rubeis et al., 2014; Fromer et al., 2014; McCarthy et al., 2014; Wen et al., 2014). These studies not only identified *de novo* causal rare variants, but also demonstrated how large their contributions are to neurodevelopmental disorders, which brings new insight into the genetic etiology. For example, according to twin studies, autism disorder has an estimated broad-sense heritability of over 0.9 (for the narrow phenotype of autism), while GWAS loci can only explain a small part of the heritability (Freitag, 2007). A recent WES study on more than 2500 simplex families showed that 12% of autism diagnoses can be explained by 13% of *de novo* missense mutations, and 9% of autism diagnoses can be explained by 43% of *de novo* likely gene-disruption mutations (Iossifov et al., 2014). Another study, using a Swedish sample, confirmed the substantial contribution of *de novo* mutations to individual autism liability, but also pointed out that population-wise, their contribution to autism liability is only 2.6%, accounting for a very modest proportion of the estimated narrow-sense heritability 52.4%, which is mostly contributed by common variation (Gauglerl et al., 2014). Notably, although contribution from *de novo* mutation to population-level phenotypic variation is small compared with common variants, *de novo* mutations are very important for individual phenotype, and thus detecting those causal *de novo* mutations is important and may lead to improvements in disease risk prediction and personalized treatment. Moreover, the fact that the detected *de novo* mutations tend to come from certain pathways further reveals the pathological mechanisms of those disorders, which may lead to novel treatment strategies.

## Statistical Methods to Detect Rare Variant Association

Effects of rare variants vary across different diseases. Even if there is a substantial contribution from rare variants, it remains challenging to detect rare variant associations due to low statistical power. Many statistical methods have been proposed to increase the signal or reduce the noise in testing variant-disease association using sequencing data. We group these methods into three general categories: group-based association test, meta-analysis, and functional annotation. However, despite using very different techniques, these three categories are closely related to each other and are often used in combination.

### Group-Based Association Tests

The major strategy used in GWAS analysis is to evaluate each SNP individually with a univariate statistic. However, standard individual variant tests are underpowered to detect rare variant effects due to the low minor allele frequency (MAF) unless effect sizes or sample sizes are very large. Moreover, rare variant association studies usually involve extreme multiple testing due to the large number of rare variants in each individual. Pelak et al. (2010) reported about 3.5 million SNVs per genome using WGS on 20 samples. This further reduces the power when type-I error is controlled. Therefore, many group-based association tests that assess the cumulative effects of multiple variants have been proposed for sequencing studies. For simplicity, we describe these strategies for the analysis of a single genomic region, e.g., a gene.

The earliest collapsing methods, also known as burden tests (e.g., CAST Morgenthaler and Thilly, 2007), collapse all rare variants in a genomic region into a single variable. This can be done either through an indicator of whether an individual has any rare variants, or through summing up the total number of rare alleles (Morris and Zeggini, 2010). Both schemes completely ignore the effect of common variants and weight all rare variants equally, independent of their allele frequency. Several weighted sum tests (WSTs) generalize these ideas and suggest weighting variants according to their frequencies (Madsen and Browning, 2009; Price et al., 2010). In this way, contributions from both common and rare variants are incorporated. Different WST approaches use different weighting schemes, but in general, they all down-weight common variants and up-weight rare ones. The variable threshold (VT) approach generalizes burden tests in another direction (Price et al., 2010). Instead of using a pre-fixed threshold for rarity, the VT approach computes the test statistics over a series of reasonable thresholds τ, and adaptively chooses the τ that maximizes the test statistic.

None of the methods discussed so far allow variants to influence the phenotype in different directions. Variants with similar MAF are also assumed to have similar effect sizes. The adaptive summation (aSUM) approach is the first method that distinguishes protective variants from deleterious ones (Han and Pan, 2010). The flexibility is further improved by kernel-based methods, e.g., sequence kernel association test (SKAT; Wu et al., 2011). Similar to the WSTs, SKAT also incorporates a weighting scheme, but both the weight and the kernel can be modified based on the prior knowledge of disease etiology. No matter what kernel or weighting scheme is used, the score test guarantees the type-I error being well-controlled. Appropriate choices would simply increase the power. Other than that, both magnitudes and directionality of the associations are estimated from data instead of pre-fixed, which again introduces great flexibility.

Burden tests and the kernel-based variance-component tests have very different model assumptions. However, Lin and Tang (2011) developed a general regression framework for rare variant association testing that unifies existing methods including WST, VT, and SKAT. Lee et al. (2012) also generalized the variance-component testing framework used in SKAT by incorporating correlation structure into the random effect so that the burden test and the original SKAT both become special cases of this general framework.

Finally, although rare variants are more likely to be causal because of selection pressure, common variants could still have substantial effects in some diseases. Therefore, it would be wise to combine the effects of common and rare variants using a statistically justified framework. Right after CAST came out, the combined multivariate and collapsing (CMC) method (Li and Leal, 2008) improved CAST by collapsing variants into subgroups based on their allele frequencies, and then applying a multivariate test. More recently, Ionita-Laza et al. (2013) used the similar idea to generalize SKAT. Different weights and kernels are chosen for common and rare variants. Then, several combination approaches can be implemented to test for the combined effect (Ionita-Laza et al., 2013).

After years of exploratory research, scientists have acquired a rich collection of methods to test for group-based association. However, each method has its unique assumptions and limitations. For example, if a large proportion of rare variants are causal at the same direction, burden tests will be the most powerful; if a genomic region consists of a mixture of deleterious and protective variants, SKAT should become the superior choice. Although general frameworks have been proposed, those models often include more parameters and use more degrees of freedom. Currently, large-scale sequencing studies are still costly, so the sample size is often not very large. Whether the general and flexible frameworks could work well in such circumstances remains to be thoroughly investigated using empirical data (Liu et al., 2014). In practice, researchers should choose the statistical method tailored to the most reasonable assumptions according to the prior knowledge of disease etiology.

### Meta-Analysis

Meta-analysis is a statistical method for pooling results from multiple independent studies. It essentially increases the sample size by incorporating summary statistics rather than relying on individual-level data from different studies, which is an important feature since individual-level data usually cannot be shared due to policies and ethical concerns. While its basic idea originated back to the 17th century (Plackett, 1970), meta-analysis is still a popular approach in biomedical research, especially in genomic studies where limited sample size is often a key limiting factor for significant discoveries. Numerous meta-analysis methods and software have been developed for GWAS [e.g., METAL (Willer et al., 2010) and GWAMA (Mägi and Morris, 2010)]. These methods have enjoyed a great success, with hundreds of GWAS meta-analyses being published (Panagiotou et al., 2013). A comprehensive comparison of these meta-analysis methods is reviewed elsewhere (Evangelou and Ioannidis, 2013).

There are three major meta-analysis strategies for individual variants: approaches based on *p*-values or *Z* scores, fixed-effects models, and random-effects models. It would be natural to extend these approaches for group-based tests in sequencing studies (**Table 1**). In fact, approaches based on *p*-values or *Z* scores can be applied to group-based association tests directly using Fisher’s or Stouffer’s methods (Fisher, 1934; Stouffer et al., 1949). However, these methods are unable to deal with the heterogeneity among studies or to estimate the overall effect size. Moreover, they have been shown to be less powerful than fixed-effects models in both simulation and real data analysis (Liu et al., 2013, 2014). In 2013, several groups independently developed score-based fixed-effects models that incorporate diverse types of group-based association tests (Lee et al., 2013; Tang and Lin, 2013; Liu et al., 2014). Traditional meta-analysis approaches for single variant associations usually involve the estimation of single-variant effect that is not stable for rare variants. Methods based on score statistics avoid this issue because it only requires fitting the null model. Another advantage of score-based procedure is that it does not require different studies to have the same set of variants. This is crucial for sequencing analysis because very rare variants are not guaranteed to exist in all the cohorts being studied. Moreover, these meta-analysis approaches have been shown to be numerically equivalent to the mega-analysis using individual-level data. Finally, Hu et al. (2013) further extended the fixed-effects models based on a key observation that the multivariate score statistic as well as the corresponding information matrix can be recovered from test statistics for single variants. This adds more flexibility into the meta-analysis framework because only statistics for individual variants need to be shared. However, this simplification is valid only under the assumption of additive mode of inheritance.

**Table 1:** A list of meta-analysis software for group-based association tests.

Name	Website	Reference
RAREMETAL	http://genome.sph.umich.edu/wiki/RAREMETAL	Feng et al. (2014)
MASS	http://dlin.web.unc.edu/software/MASS	Tang and Lin (2013)
MetaSKAT	http://www.hsph.harvard.edu/xlin/software.html	Lee et al. (2013)
MAGA	http://web1.sph.emory.edu/users/yhu30/software.html	Hu et al. (2013)

Fixed-effects models assume the genetic effects to be the same in different studies. In contrast, random-effects models test for the heterogeneous genetic associations by allowing the genetic effects to vary across studies. Traditional random-effects meta-analysis models test for the mean effect of genetic variables (DerSimonian and Laird, 1986). Although it reflects the heterogeneous nature, this type of methods tend to be less powerful than fixed-effects models (Evangelou and Ioannidis, 2013). Han and Eskin (2011) improved the discovery power of random-effects models by testing for the joint null hypothesis of the absence of any genetic effects and between-study variance. Tang and Lin (2014) extended the same idea to random-effects group-based meta-analysis while using the score-based framework. Lee et al. (2013) also proposed a random-effects model. It demonstrated comparable discovery power in simulations compared to the method developed by Tang and Lin (2014).

### Functional Annotation

Genomic functional annotation is crucial for prioritizing variants and interpreting results in association studies. This is especially helpful for predicting causal variants among a group of SNVs with strong LD. With the help of appropriate annotation tools, both random and systematic noise in the data can be greatly reduced. There are some techniques that use special study design or simple filtering rather than statistical models to incorporate functional annotations. For example, in terms of study design, we have discussed that WES is sometimes more preferred than WGS. One consideration is that variants in the protein-coding regions are more likely to be functional. In terms of variant-filtering procedures, various pipelines have been developed to focus on a certain type of variants such as non-synonymous SNPs or frame-shifting insertions and deletions (Vissers et al., 2010; Xu et al., 2011; Kim et al., 2012; Reumers et al., 2012). These variant filters suffer from a high chance of missing real causal candidates by enforcing variants to satisfy every screening condition. In contrast, well-justified statistical methods allow incorporating diverse types of information to collectively evaluate the functional potential of variants. Here, we focus on the statistical tools that predict functional genomic variants.

Methods for predicting deleterious variants in the protein-coding regions are the richest of the available approaches. Numerous tools have been developed to serve this purpose, including SIFT (Ng and Henikoff, 2001), PolyPhen (Ramensky et al., 2002; Adzhubei et al., 2010), MutationTaster (Schwarz et al., 2010), SAPRED (Ye et al., 2007), and SNPs3D (Yue et al., 2006), among others. Most of these methods are statistical classifiers using both evolutionary and biochemical information of proteins as annotation features (Cooper and Shendure, 2011). The major differences among these tools are the choices of training data, covariates, and classification methods. Compared with the “mysterious” non-coding regions in the human genome, researchers have gained a much deeper understanding of the protein-coding regions through tracing the functional mechanisms in transcription and translation. Therefore, it is not surprising that some covariates (e.g., amino acid properties and protein structural information) are informative for predicting deleteriousness of variants in coding regions. The positive training data are usually collected from large databases for pathogenic variants [e.g., OMIM (Hamosh et al., 2005) and ClinVar (Landrum et al., 2014)], while some matched benign variants are used as the negative training set. Finally, statistical classification methods (e.g., naïve Bayes classifier and support vector machine) are trained on the training data using collected covariates. The informative covariates, the gold-standard training data, and the statistically justified classification frameworks altogether guarantee the predictive ability of these tools.

Compared to the well-understood coding regions, the non-coding regions in the human genome are much less explored. However, it has been established that ∼98% of the human genome is non-coding DNA (Elgar and Vavouri, 2008). About 95% of known variants within sequenced genomes and nearly 90% of the significant variants from GWAS lie outside of protein-coding regions (Hindorff et al., 2009). All these pieces of evidence, as well as the expected wide applications of WGS in the near future, suggest that the scope of the annotation tools should be extended to the whole-genome. Several tools have been developed, including HaploReg (Ward and Kellis, 2012a), RegulomeDB (Boyle et al., 2012), CADD (Kircher et al., 2014), and GWAVA (Ritchie et al., 2014; **Table 2**). Among these tools, HaploReg and RegulomeDB are more of databases than prediction tools. They both offer well-designed user interfaces that present many useful annotation data collected from different sources such as the ENCODE project (Dunham et al., 2012). The users need to judge the functional potential of variant candidates based on these annotations by themselves. CADD and GWAVA are similar to the deleteriousness prediction tools developed for coding regions. CADD is based on support vector machine, while GWAVA uses the random forest algorithm. Large consortia such as the ENCODE project have generated a vast amount of regulatory information for the human genome (Dunham et al., 2012). Among those, information of the transcriptional binding sites, histone modification, DNase I hypersensitivity, DNA methylation, and many others all have the potential to serve as predictive covariates in non-coding functional annotation tools. However, current supervised-learning-based methods still suffer from potentially biased training data due to our limited knowledge of non-coding functional mechanism. Therefore, methods based on unsupervised learning may be advantageous at this early stage, but no such method has been proposed yet.

**Table 2:** A list of tools for annotating variants in non-coding regions.

Name	Website	Reference
HaploReg	http://www.broadinstitute.org/mammals/haploreg	Ward and Kellis (2012a)
RegulomeDB	http://regulome.stanford.edu	Boyle et al. (2012)
FunSeq2	http://funseq2.gersteinlab.org	Fu et al. (2014)
GWAVA	http://www.sanger.ac.uk/sanger/StatGen_Gwava	Ritchie et al. (2014)
CADD	http://cadd.gs.washington.edu	Kircher et al. (2014)
FATHMM-MKL	http://fathmm.biocompute.org.uk	Shihab et al. (2015)
Phen-Gen	http://phen-gen.org	Javed et al. (2014)

Integrating functional annotations in causal variant detection is a very active research field with many challenging open questions. While this review was in preparation, a new non-coding variant functional prediction method based on multiple kernel learning was published (Shihab et al., 2015). Here we only introduced some of the existing tools that are closely related to sequencing study. It is worth noting that researchers should choose the most appropriate annotation tool based on the scientific hypothesis. For example, in cancer studies, methods that predict regulatory somatic mutations will probably be favored (Khurana et al., 2013; Fu et al., 2014). When the phenotypic information is available, methods that integrate phenotype-specific gene prioritization may be advantageous (Sifrim et al., 2013; Javed et al., 2014; Singleton et al., 2014).

## Discussion

In the last 10 years, GWAS has transformed genetic epidemiology to genomic epidemiology. More than 2,000 GWAS have been done for almost all known complex diseases, leading to the identification of a vast number of disease-associated genomic loci. Despite these discoveries, more and more people have realized the limitations of this experiment design. In this review, we discussed some of the well-known issues in GWAS analysis, including missing heritability and lack of interpretability. Recent advances of the next generation sequencing technology have made sequencing faster, more affordable and more accurate. These technological advances as well as the success of pioneer sequencing studies strongly suggest that NGS has the potential to lead genomic epidemiology into a new era. It allows systematic assessment of rare SNVs as well as many other diverse types of genomic and epigenetic variations using hypothesis-free whole-genome scans. Large consortia and programs have also been formed, e.g., the 1000 Genomes Project Consortium (Genomes Project et al., 2012) and the NHGRI Genome Sequencing Program (GSP), in this mission of decoding the variations of human genome using NGS technologies. All these advances would bring biological insight and benefit scientific researches.

Besides its benefits to the basic science, NGS also has a bright future in clinical applications. For example, WES identified a missense mutation in a 15-months child patient with symptoms similar to Crohn’s disease. Based on this, the child received proper diagnosis and treatment, which would otherwise be intractable (Worthey et al., 2011). Notably, clinical cases like this also benefit scientific research as novel causes of disorders are revealed in the process. Programs that use NGS to aid diagnosis have been launched, e.g., “3Gb-testing” project (Boccia et al., 2014). As sequencing technologies become more mature and affordable, we expect the potential of NGS to be fully realized as a bridge between clinical applications and research progresses.

Although the future of NGS is very promising, many challenges still remain. First, although the cost of generating sequencing data continues to drop, it is still substantially greater than the cost of more traditional technologies. Currently, sequencing cost ranges from 500 (70-fold WES) to 1000 dollars (30-fold WGS) per sample, which is nearly 10 times greater than using high-quality microarrays. Apart from the cost of data generation, the demanding requirement of sample recruitment, data storage, and downstream processing all act as barriers to sample size and statistical power (Sboner et al., 2011). Moreover, some issues such as the optimal combination of sequencing depth and sample size can only be answered using empirical data, and are still far from being fully understood. When designing NGS-based studies, researchers should take all these factors into consideration in order to choose the most appropriate study design. Finally, finding causal variants from the overwhelmingly large number of background mutations is a great challenge. In large population-based WGS studies, the number of SNVs that appear at least once can easily go beyond 10 million. This leads to extreme multiple testing problems for which any traditional statistical procedure is likely to be underpowered. We have discussed several categories of novel statistical methods designed for sequencing analysis, including group-based association tests, meta-analysis approaches, and annotation tools for variant prioritization. But these do not cover all aspects of statistical methods that can be used in sequencing studies. With the popularization of next generation sequencing, we expect to see a boom in novel and powerful statistical approaches, amazing scientific discoveries, as well as clinical breakthroughs.

## Author Contributions

QW and QL conceived and wrote the paper. HZ advised on statistical and genetic issues.

## Conflict of Interest Statement

The authors declare that the research was conducted in the absence of any commercial or financial relationships that could be construed as a potential conflict of interest.
